# Graph Embedding Deep Learning Guides Microbial Biomarkers' Identification

**DOI:** 10.3389/fgene.2019.01182

**Published:** 2019-11-22

**Authors:** Qiang Zhu, Xingpeng Jiang, Qing Zhu, Min Pan, Tingting He

**Affiliations:** ^1^School of Information Management, Central China Normal University, Wuhan, China; ^2^School of Computer, Central China Normal University, Wuhan, China; ^3^Hubei Provincial Key Laboratory of Artificial Intelligence and Smart Learning, Central China Normal University, Wuhan, China

## Abstract

The microbiome-wide association studies are to figure out the relationship between microorganisms and humans, with the goal of discovering relevant biomarkers to guide disease diagnosis. However, the microbiome data is complex, with high noise and dimensions. Traditional machine learning methods are limited by the models' representation ability and cannot learn complex patterns from the data. Recently, deep learning has been widely applied to fields ranging from text processing to image recognition due to its efficient flexibility and high capacity. But the deep learning models must be trained with enough data in order to achieve good performance, which is impractical in reality. In addition, deep learning is considered as black box and hard to interpret. These factors make deep learning not widely used in microbiome-wide association studies. In this work, we construct a sparse microbial interaction network and embed this graph into deep model to alleviate the risk of overfitting and improve the performance. Further, we explore a Graph Embedding Deep Feedforward Network (GEDFN) to conduct feature selection and guide meaningful microbial markers' identification. Based on the experimental results, we verify the feasibility of combining the microbial graph model with the deep learning model, and demonstrate the feasibility of applying deep learning and feature selection on microbial data. Our main contributions are: firstly, we utilize different methods to construct a variety of microbial interaction networks and combine the network *via* graph embedding deep learning. Secondly, we introduce a feature selection method based on graph embedding and validate the biological meaning of microbial markers. The code is available at https://github.com/MicroAVA/GEDFN.git.

## graph embedding, deep learning, feature selection, biomarkers, microbiomeIntroduction

A large number of microorganisms are parasite on various parts of the human body, mainly concentrated in the intestine, oral cavity, reproductive tract, epidermis and skin. The microbial communities existing in different parts of the body or in different host environments are very different (Turnbaugh et al., 2007; Lloyd-Price et al., 2017). These microorganisms include bacteria, fungi, viruses and protozoa. All genetic material in the particular microbial community is called the microbiome. Recent studies have shown that microorganisms are directly or indirectly related to many diseases. For example, the gut microbiome may be closely related to irritable bowel syndrome and its imbalance may lead to chronic kidney diseases. Microorganisms may also be closely related to digestive tract diseases, endocrine diseases, circulatory diseases, reproductive system diseases, respiratory and psychiatric diseases (Kho and Lal, 2018). Since the microbiome plays a central role in the hosts' health, understanding the distribution and composition of microbial communities in humans, especially under different diseases or physiological conditions, is of great significance for disease diagnosis, prevention and treatment. The microbiome-wide association studies are to find disease-associated microbial markers to guide disease diagnosis and treatment (Gilbert et al., 2016; Wang and Jia, 2016). Compared with the human genome, the microbiome is an ideal target and more convenient to regulate. Therefore, the microbiome is often named “the second human genome” (Brüls and Weissenbach, 2011). However, there are many types of microorganisms and most of them cannot be cultured. Therefore, a high-throughput sequencing method is a feasible means of understanding microbial communities. Through high-throughput sequencing, we can understand the types of microorganisms and even their functions in the community (Ranjan et al., 2016).

The microbiome data is from high-throughput sequencing methods such as 16s or shotgun sequencing, which is often with high dimensions with noise. As a result, it is difficult to mine microbial signatures from these data. Traditionally, statistical-based methods identify markers mainly through microbial abundance differential expression (Paulson et al., 2013). However, the statistical approaches often have strong assumptions and the real data often do not satisfy these assumptions (Hawinkel et al., 2017; Weiss et al., 2017). Other machine learning methods are widely explored (Pasolli et al., 2016). Recently, deep learning has received great attention, especially its end-to-end automatic learning ability. At present, deep learning is widely used in automatic driving, image recognition and text processing, which has received exciting results (LeCun et al., 2015). The deep models can learn specific patterns directly from the data, thus avoiding the artificial feature engineering (Goodfellow et al., 2016; Kong and Yu, 2018). In the analysis of biomedical data, especially the analysis of various omics data, deep learning has achieved good improvement, but still faces many problems and challenges (Angermueller et al., 2016; Camacho et al., 2018; Eraslan et al., 2019). First, deep learning requires a large amount of training data to learn useful information while the biological sample size is often limited and cannot fully utilize its capabilities. Second, the training process is often considered a black box and people can only control the input and models' parameters. More specifically, deep learning involves complex network structures and nonlinear transformations, as well as a large number of hyperparameters, which hinder people from understanding how deep neural networks are making predictions. Although deep neural networks perform well on some classification tasks, biological problems should be paid more attention to which features lead to better classification (Ching et al., 2018).

In this paper, we propose a feature selection method based on Graph Embedding Deep Feedforward Network (GEDFN) to conduct microbiome-wide association studies. Firstly, we construct three different microbial co-occurrence interaction networks. We utilize a graph embedding method to embed the network as *a priori* knowledge into Deep Feedforward Neural Network to reduce parameters, alleviate the overfitting problem and improve the models' performance. Secondly, we propose a feature selection approach based on GEDFN. Experiments show the microbial feature markers obtained *via* this method have biological significance. In other words, our results demonstrate graph embedding deep learning could guide feature selection.

## Related Work

### Microbial Interaction Network

Because of the various relationships between microorganisms, such as symbiosis, competition and so on, as well as the complex structure and function of microorganisms due to their dynamic properties, the network is a good way to represent complex relationships. Understanding microbial interaction can help us understand microbial functions. System-oriented graph theory can facilitate microbial analysis and enhance our understanding of complex ecosystems and evolutionary processes (Faust et al., 2012; Layeghifard et al., 2017). However, most microorganisms are uncultured, we can only construct microbial interaction networks from high-throughput sequencing data. At present, there are many computational methods to construct microbial interaction networks. In theory, any method of calculating features' relationships can be used. For example, Bray–Curtis can be used to measure species abundance similarity (Bray and Curtis, 1957). The Pearson correlation coefficient is used to evaluate the linear relationship and the Spearman correlation coefficient can measure the rank relationship (Mukaka, 2012). CoNet uses an ensemble approach and combines with different comparison metrics to detect different relationships (Faust and Raes, 2016). Maximum mutual information is designed to capture broader relationships, not limited to specific function families (Reshef et al., 2011). MENA applies random matrix theory to conduct microbial analysis and experiments show it is robust to the noise and threshold (Deng et al., 2012). Sparse Correlations for Compositional data (SparCC) is a tool based on Aitchison's log ratio transformation to conduct microbial composition analysis (Friedman and Alm, 2012). SParse InversE Covariance Estimation for Ecological Association Inference (SPIEC-EASI) combines data logarithmic transformation with graph model inference framework to build a correlation network (Kurtz et al., 2015).

### Feature Selection

Real biomedical data, especially various omics data with high dimensions and noise, often has feature redundancy problem. Feature selection is a step of data preprocessing, which involves selecting related features from a large number of features to improve subsequent learning tasks (Li et al., 2017).

There are mainly three kinds of feature selection methods, including filter, wrapper and embedded method. The filter approach selects subset features and then trains the learner. The feature selection process is independent of the subsequent learner. This is equivalent to filter the initial feature with the feature selection process and train the model with the filtered features. However, filter methods often ignore some features that are helpful for classification. At the same time, many filter methods are based on a single-featured greedy algorithm. The assumption is that each feature is independent while this is often not the case in microbiological data. The wrapper feature selection directly takes the performance of the learner to be used as the evaluation criterion of the feature subset. In other words, the purpose of the wrapper feature selection is to select a feature subset that is most efficient in its performance for a given learner. Compared to the filter method, the wrapper method can evaluate the result of feature selection to improve the classification performance; however, the feature selection process requires to train the learner iteratively and the calculation is huge (Li et al., 2017). The embedded feature selection combines the feature selection in the learning and training process, both of which are completed in the same optimization. In other words, the feature selection is automatically performed during the training.

Feature selection is a traditional machine learning research field with many methods. For more information, please refer to the literature (Li et al., 2017). The previous work proposed a feature selection method based on Deep Forest (Zhu et al., 2018); however, there is less work on microbiome-wide association studies *via* Deep Neural Network and less research is done from the perspective of embedding approach for feature selection. The challenge of feature selection based on microbial network is that there is no microbial network available at present. The commonly used statistical-based interaction network methods may lead to high false positive rate due to the compositional bias (Gloor et al., 2017).

## Materials and Methods

**Figure 1:**
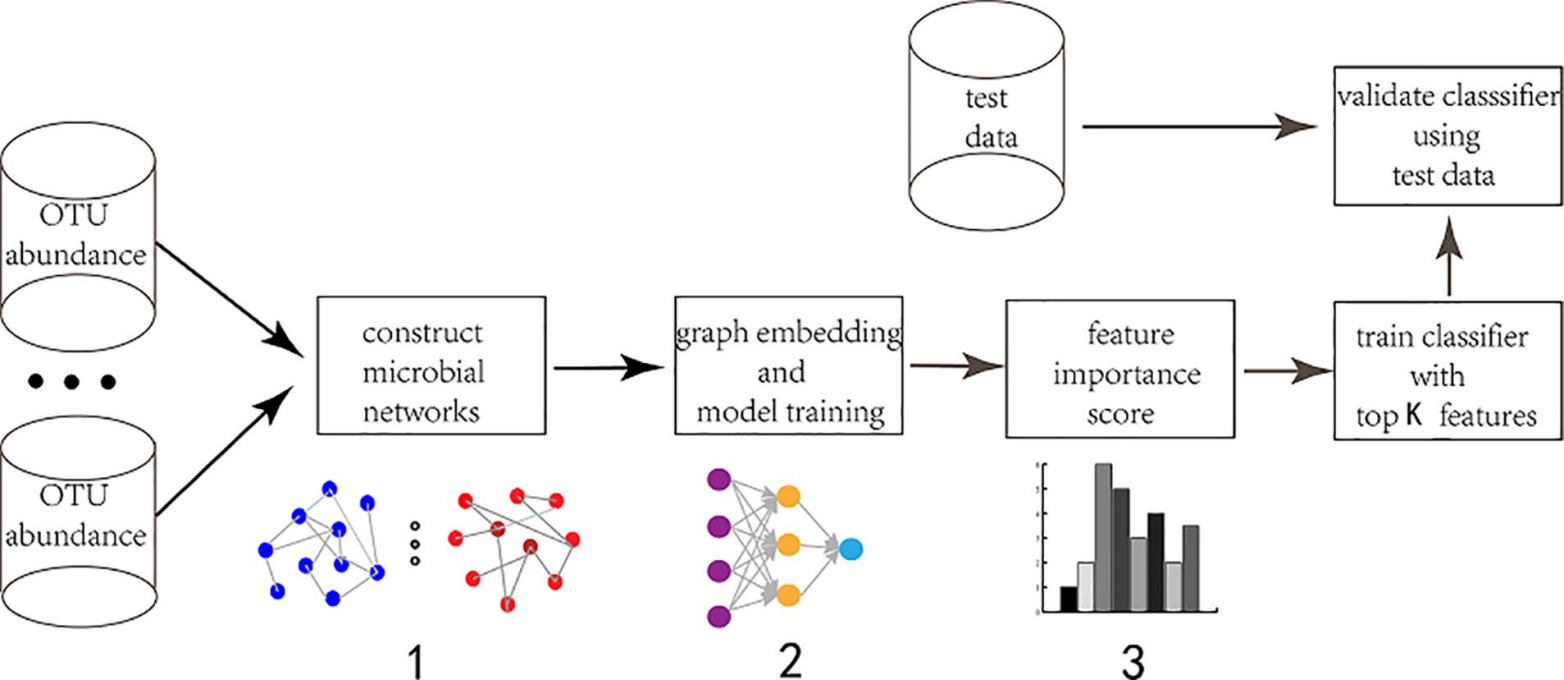
The workflow of graph embedding deep network to conduct feature selection. 1. Construct microbial interaction network. The input is Operational Taxonomic Unit (OTU) abundance. Different approaches are adopted to obtain different interaction networks. The vertexes are species and the edges are correlation coefficient. 2. Graph embedding and model training. The feature graph is embedded into the first hidden layer in order to achieve sparse connection instead of fully-connected between the input layer and the first hidden layer. The first hidden layer (graph embedding layer) has the same neurons as the input layer. 3. Feature selection. The neurons (features) are ranked according to their importance score which is calculated *via* each neuron's connection weights.

We mainly explain the feature selection method based on GEDFN from the following three aspects (**Figure 1**). First, we will introduce the construction method of microbial interaction network, including sparcc, SPIEC-EASI and Maximal Information Coefficient (MIC) then, we will introduce a deep embedding structure to embed the graph into Deep Feedforward Network. Finally, we will propose a feature selection approach for GEDFN.

### Microbial Correlation Network

The total amount of genetic material extracted from the microbial community and the sequencing depth will affect the whole reads. It is often necessary to normalize the reads in the sample. As a result, the microbial abundance obtained by 16s sequencing is relative rather than absolute, which is not independent. The traditional statistical measures for detecting microbial interactions, for example, Pearson correlation, will lead to false positives (Gloor et al., 2017).

#### Sparcc

Assuming that the network is sparse, sparcc constructs the association network by using standard logarithmic ratio transformation and iteratively calculates the variance matrix of compositional dependence. For details of the algorithm, please refer to the literature (Friedman and Alm, 2012).

#### SPIEC-EASI

SPIEC-EASI assumes the network is sparse and combines logarithmic transformation of compositional data with graph inference framework to construct the network. It consists of two steps: first, logarithmic ratio transforms the data; then, SPIEC-EASI uses the neighborhood selection and sparse inverse covariance selection to infer the interaction graph from the transformed data (Kurtz et al., 2015).

#### Maximal Information Coefficient

The maximal information coefficient (MIC) is used to measure the degree of linear and nonlinear correlation between two variables (Reshef et al., 2011). The main idea of the MIC method is based on the recognition that if there is some correlation between two variables, the distribution of the data in the grid can be reflected after meshing the scatter plots formed by the two variables. The MIC divides the scatter plot of the variable pair (x, y) and uses dynamic programming to calculate and search for the maximum mutual information value that can be achieved under different split modes. Finally, the maximum mutual information value is normalized and the result is MIC.

### The Framework of Graph Embedding Deep Feedforward Network

#### Deep Feedforward Neural Network

Deep Feedforward Network, also known as feedforward neural network or multilayer perceptron, is a typical deep learning model. In this model, the information moves only in one direction from the input nodes to the output nodes through the hidden nodes. There is no loop in the network. A feedforward neural network structure with *l* hidden layers is:

(1) $$\text{P}\left(\text{y|X},\theta \right)=f\left(Z_{out}W_{out}+b_{out}\right)$$

(2) $$Z_{out}=\;\sigma \left(Z_{l}W_{l}+\;b_{l}\right)$$

(3) $$Z_{k+1}=\;\sigma \left(Z_{k}W_{k}+\;b_{k}\right)$$

(4) $$\;\;\;\;\;\;\;Z_{1}=\;\sigma \left(XW_{in}+\;b_{in}\right)\;\;\;\;\;\;\;$$

where X∈*R* *^nxp^* is an input matrix with *n* samples and *p* features, y∈*R* *^n^* is the output label for the classification task. In this work, it is a binary classification. The label for each sample is normal or disease. Z*_out_* and Z*_k_* *,*(*k*=1,…,*l*-1) are the neurons in the hidden layer. W*_k_* is the weight matrix. b*_k_* is the bias. θ is the parameters. *σ*(·)is the activation function(such as, sigmoid, tanh, rectifiers). *F*(·) is a softmax function which is used to convert the output layer value into the predicted probability.

The model uses a stochastic gradient descent (SGD) algorithm to minimize the cross entropy loss function to update the parameter *θ*. When a feedforward neural network is used to receive input x and produce an output $\hat{\text{ y }}$ . During training, forward propagation can continue until it produces a scalar cost function *J*(*θ*). The backpropagation algorithm runs information from the cost function and flow backward through the network to calculate the gradient in order to update the weight parameters (Goodfellow et al., 2016).

(5) $$J\left(\theta \right)=\;-\frac{1}{n}\sum_{i=1}^{n}\left(y_{i}log{\hat{p}}_{i}+\left(1-y_{i}\right)\text{log}\left(1-{\hat{p}}_{i}\right)\right)$$

#### Graph Embedding Deep Feedforward Network

**Figure 2:**
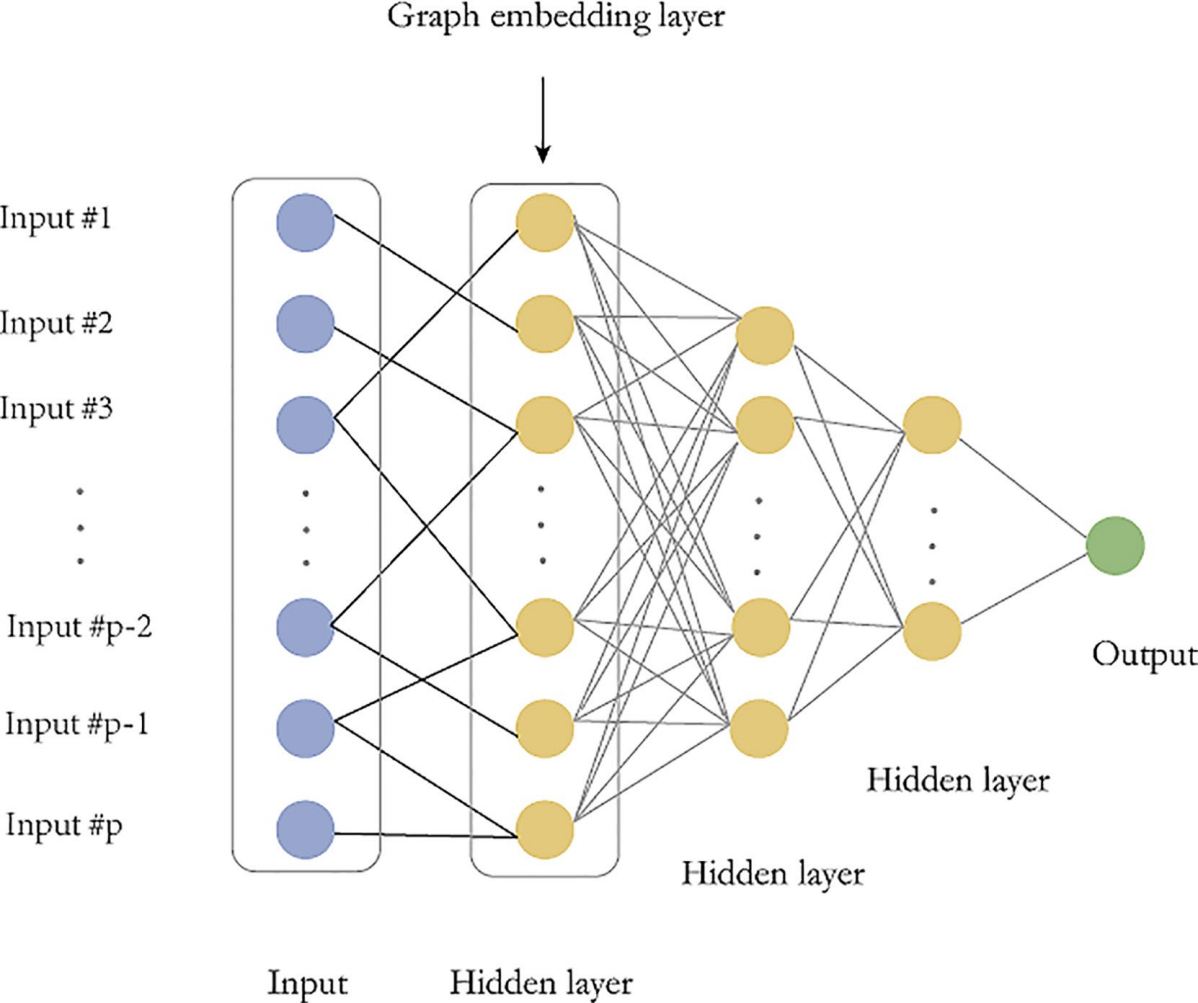
Graph embedding deep feedforward network (GEDFN). The graph embedding layer (first hidden layer) has same neurons with the input layer. The sparse connect between the input layer and the first hidden layer is marked as black. Other hidden layers are fully-connected.

The fully connected deep feedforward neural network has many parameters and requires a large number of training data, but often the biological sample size is limited, which often leads to overfitting. Therefore, we construct a microbial sparse network and embed this graph network into the model. There are two main advantages. First, the sparse graph embedding will greatly reduce the parameters of deep feedforward network and mitigate the overfitting risk. Second, the sparse graph structure is derived from existing prior information and combining the priori information into the network can improve the reliability of the model. The main idea of graph embedding is to replace the full connections between the input layer and the first hidden layer with a sparse graph (**Figure 2**).

Consider a graph G=(V,E), V is the vertical set with *p* features. E is a collection of all edges. A common way of representing a graph is to use an adjacency matrix. Given a graph G with *p* vertices, a *pxp* adjacency matrix A is:

$$A_{ij}=\{\begin{array}{c} 1,\;if\;{\text{V}}_{\text{i}}\;and\;{\text{V}}_{\text{j}}\;connected,\;\forall i,j=1,\ldots ,p\\ 0,\;\;\;\;\;\;\;\;\;\;\;\;\;\;\;\;\;\;\;\;\;\;\;\;\;\;\;\;\;\;\;\;\;\;\;\;\;\;\;\;\;\;\;\;\;\;\;\;\;\;\;\;\;\;\;\;\;otherwise. \end{array}$$

G is an undirected graph and A is a symmetric matrix. At the same time, we consider *A* *_ii_*=1 which indicates that the vertex itself is connected. We construct a feedforward neural network in which the first hidden layer has the same dimensions as the input layer, *h* *_in=_* *p*, similarly,*W* *_in_* is a *pxp* matrix. The input X is sparsely connected with Z_1_ (**Figure 2**). In other words, the original fully connected layer:

(6) $$Z_{1}=\sigma \left(XW_{in}+b_{in}\right)$$

is changed to:

(7) $$Z_{1}=\sigma \left(X\left(W_{in}⊙A\right)+b_{in}\right)$$

Where  ⊙ is element-wise product. Therefore, the connection between the input and first hidden layer of the feedforward network is filtered by the graph adjacency matrix. Each feature is corresponding to a hidden neuron. All features have corresponding hidden neurons in the first hidden layer. The feature can only provide information to the connected graph. In this way, the graph helps to achieve the sparsity of the connection between the input layer and the first hidden layer (Kong and Yu, 2018).

### Feature Selection Based on GEDFN

In addition to improving classification, it is also meaningful to find features that contribute significantly to classification because they reveal potential biological mechanisms. However, Deep neural network is a “black box”, the interpretability of deep learning hasn't been well-defined (Guidotti et al., 2019). In our experiment, we focus on how the input features influence the prediction and we borrow the idea from Olden and Jackson (2002) and Kong and Yu (2018). The feature importance score is the quantification values of the contributions of features to a model prediction, which links the input features and output prediction. They highlight the parts of a given input that are most influential for the model prediction and thereby help to explain why such a prediction was made. The feature selection is based on feature score, which means the score is high if the feature is important. As a result, we develop a feature ranking method based on the feature relative importance score, similar to the connection weights method introduced by Olden and Jackson (2002) and Kong and Yu (2018). What is learned by neural networks is contained in the connection weights. Based on idea of connection weight, we propose a graphical connect weight method that emphasizes the importance of the features of our proposed neural network architecture.

The main idea of a graphical connect weight is: the contribution of a particular variable directly reflects the magnitude of the connection weights associated with the corresponding hidden neurons in the graph embedding layer. The sum of the absolute values of the directly related weights for a neuron (or feature) gives its relative importance:

(8) $$s_{j}=\gamma_{j}\sum_{k=1}^{p}|w_{kj}^{\left(in\right)}I(A_{kj}=1)|+\Sigma_{m=1}^{h_{1}}\left|w_{jm}^{\left(1\right)}\right|,$$

(9) $$\gamma_{j}=\min\left(c/\sum_{k=1}^{p}\left(A_{kj}=1\right),1\right),\;j=1,\;\ldots ,p.$$

Where s*_j_* is importance score of the feature *j w* ^(^ *^in^* ^)^ indicates the weights between the input layer and the first hidden layer, while w^(1)^ indicates the weights between the first and second hidden layer. The constant *c* is to penalize vertices with too many connections so that they don’t over impact the result. In the following experiments, we set the parameter *c *= 50.

## Experiments and Results

### Data Set

Inflammatory bowel diseases (IBD) are a group of specific chronic intestinal diseases, mainly including Crohn's disease and ulcerative colitis. The occurrence and development of IBD are closely related to intestinal microorganisms (Gevers et al., 2014). In our experiment, OTU BIOM files and metadata were downloaded from the QIITA (https://qiita.ucsd.edu/) database (study id: 1939). The detailed experiment was described in Gevers et al., 2014. The IBD data set consists of 1,359 metagenomic samples, including rectal, ileal biopsy and fecal samples (Gevers et al., 2014). We retained samples of mucosal tissue biopsies (terminal ileum and rectum) samples under the age of 18. The control group were without inflammatory conditions, such as abdominal pain and diarrhea. The final data set consisted of 657 IBD samples and 316 normal samples, respectively. We used QIIME's taxa collapse to filter the strain's species, limiting features at genus level.

### Results

#### The Hyperparameters of Graph Embedding Deep Feedforward Neural Network

The structure of the graph embedding deep feedforward neural network (GEDFN) is shown in Figure 2. The most important part of GEDFN is that the number of neurons in the first hidden layer is the same as the number of neurons in the input layer and they are sparsely connected, which is different with normal fully connected feed forward neural network. The second layer, third and fourth hidden layers are consisting of 128, 64 and 16 neurons respectively and they are fully connected.

We use three different methods to construct a microbial co-occurrence interaction network from microbial abundance data. When the sparcc method is used to build the network, we reserve the vertexes if the correlation of two vertexes is larger than 0.3. We get an adjacency network with 63 vertexes and 315 edges. We adopt the mictools (Albanese et al., 2018) to build the MIC relevant network and we get 279 vertexes and 3230 edges when the correlation threshold is 0.2. The network constructed by sparcc and SPEC-EASI methods is sparse while MIC gets relatively a dense network. Different methods get different interaction networks. We find the higher the threshold, the more reliable is the network. However, the high threshold will make the network too sparse. As a result, we combine three kinds of networks to get a larger network with 736 vertexes and 18,034 edges. In this way, the connections between the input layer and the first hidden layer are more reliable and less dense than the fully connected approach.

Other hyperparameters of GEDFN are as follows: the learning rate is 0.0001, the activation function is Rectified Linear Unit (ReLU) and the weight initializer is he_uniform, the drop out is 0.2. the code is implemented in keras and available at https://github.com/MicroAVA/GEDFN.git. 

#### The Evaluation of Classification

**Figure 3:**
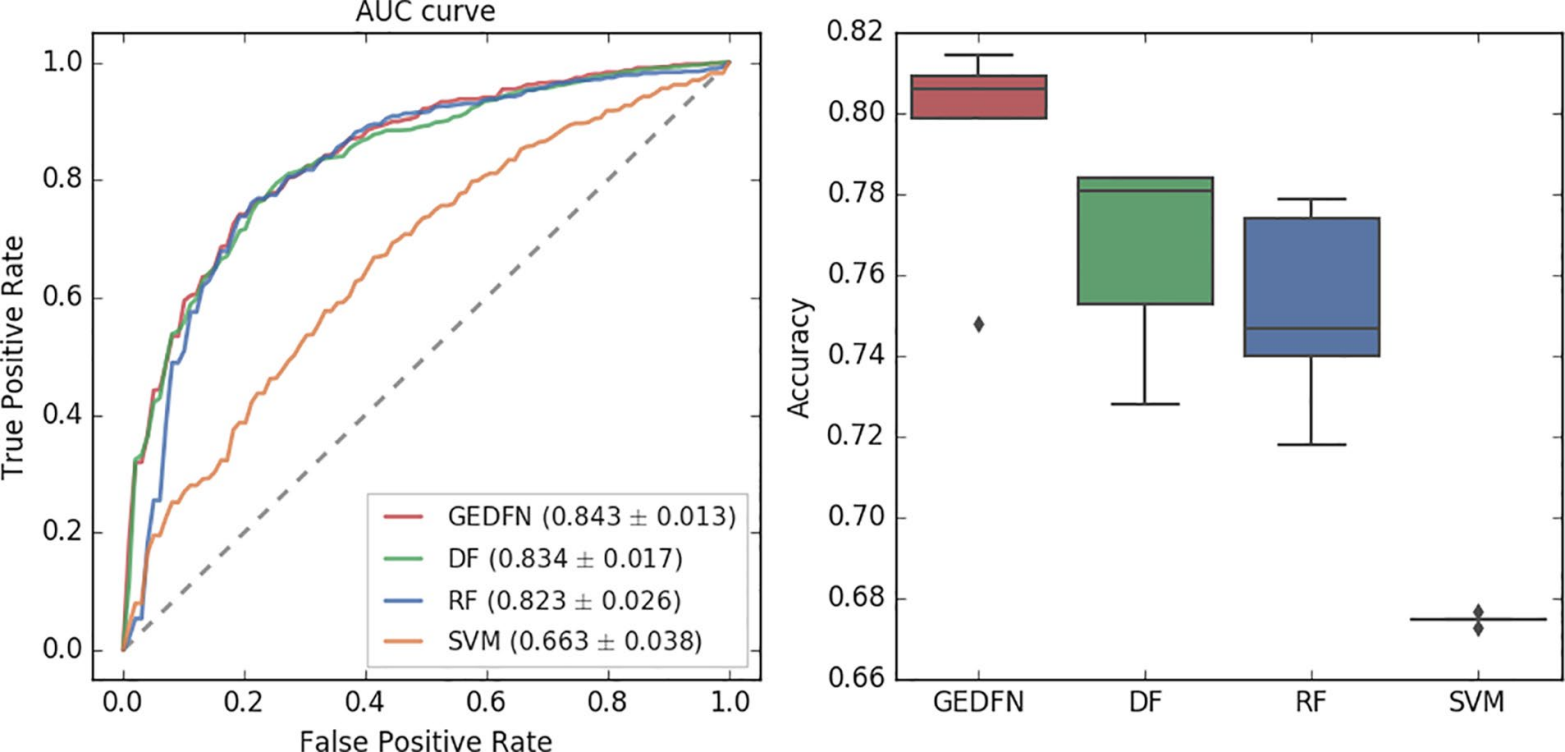
The Area Under Receiver Operating Characteristic curve (left) and accuracy of classification (right) for GEDFN, Deep Forest (DF), Random Forest (RF) and Support Vector Machines (SVM). Left: the grey dash line is the chance discrimination that located on diagonal line (AUC = 0.5). The maximum AUC = 1 means the classifier could discriminate the diseased and non-diseased perfectly while AUC = 0 means the classifier incorrectly classified all subjects with diseased as negative and all subjects with non-diseased as positive. The AUC is averaged through a five-fold cross validation. Right: the boxplot for classifiers’ classification accuracy.

Traditional classification methods such as Random Forest has been shown to be the best performers in omics data classification tasks and the results show that Random Forest has achieved the best performance on microbial classification (Pasolli et al., 2016). Therefore, we compare GEDFN with Deep Forest (DF), Random Forest (RF) and Support Vector Machines (SVM). For the binary classification, we calculate the Area Under the Receiver Operating Characteristics (AUROC) and classification accuracy for each method (**Figure 3**).

AUROC curve is a performance measurement for classification problem at various thresholds settings, which can evaluate classifiers considering all true positives (TP), false positives (FP), true negatives (TN) and false negatives (FN). Receiver Operating Characteristics (ROC) is a probability curve and Area Under the Curve (AUC) represents degree or measure of separability. It tells how much a model is capable of distinguishing between classes. The higher the AUC, the better the model is at predicting 0s as 0s and 1s as 1s. By analogy, the higher the AUC, the better the model is at distinguishing between patients with disease and no disease. The ROC curve is plotted with true positive rate (TPR) against the false positive rate (FPR) where TPR is on the y-axis and FPR is on the x-axis.

$$\text{TPR}=\frac{TP}{TP+FN}\;,\;FPR=\;\frac{FP}{TN+FP}$$

The classification accuracy means the percentage of correct predictions from the total number of predictions made.

$$\text{ACC}=\;\frac{1}{m}\overset{m}{\underset{i=1}{\sum }}I\left(\hat{y}=y\right)$$

Where $\hat{y}$ is the predicted label and *y* *_i_* is the true label for the sample *i*. The *m* means the sample size and *I*(·)is the indicator function.

In this experiment, we adopt a five-fold cross-validation. We use the implementation of Random Forest in python's scikit-learn package. We set the estimator parameter to 300. The Deep Forest is based on the work (Zhu et al., 2018). From the AUC value, we find that the Graph Embedding Deep Feedforward Network (GEDFN) is much better than SVM (AUC = 0.663). Compared with Deep Forest and Random Forest, GEDFN is also very competitive. GEDFN achieves an AUC value of 0.843, which is slightly better than Deep Forest (AUC = 0.834) and Random Forest (AUC = 0.823). In terms of classification accuracy, GEDFN achieves an average accuracy of 79.52%, Deep Forest achieves 76.6% and Random Forest achieves 75.16%. GEDFN outperforms 2–4% than Deep Forest and Random Forest. These methods are much better than SVM (67.5%).

#### The Evaluation of Feature Selection

**Figure 4:**
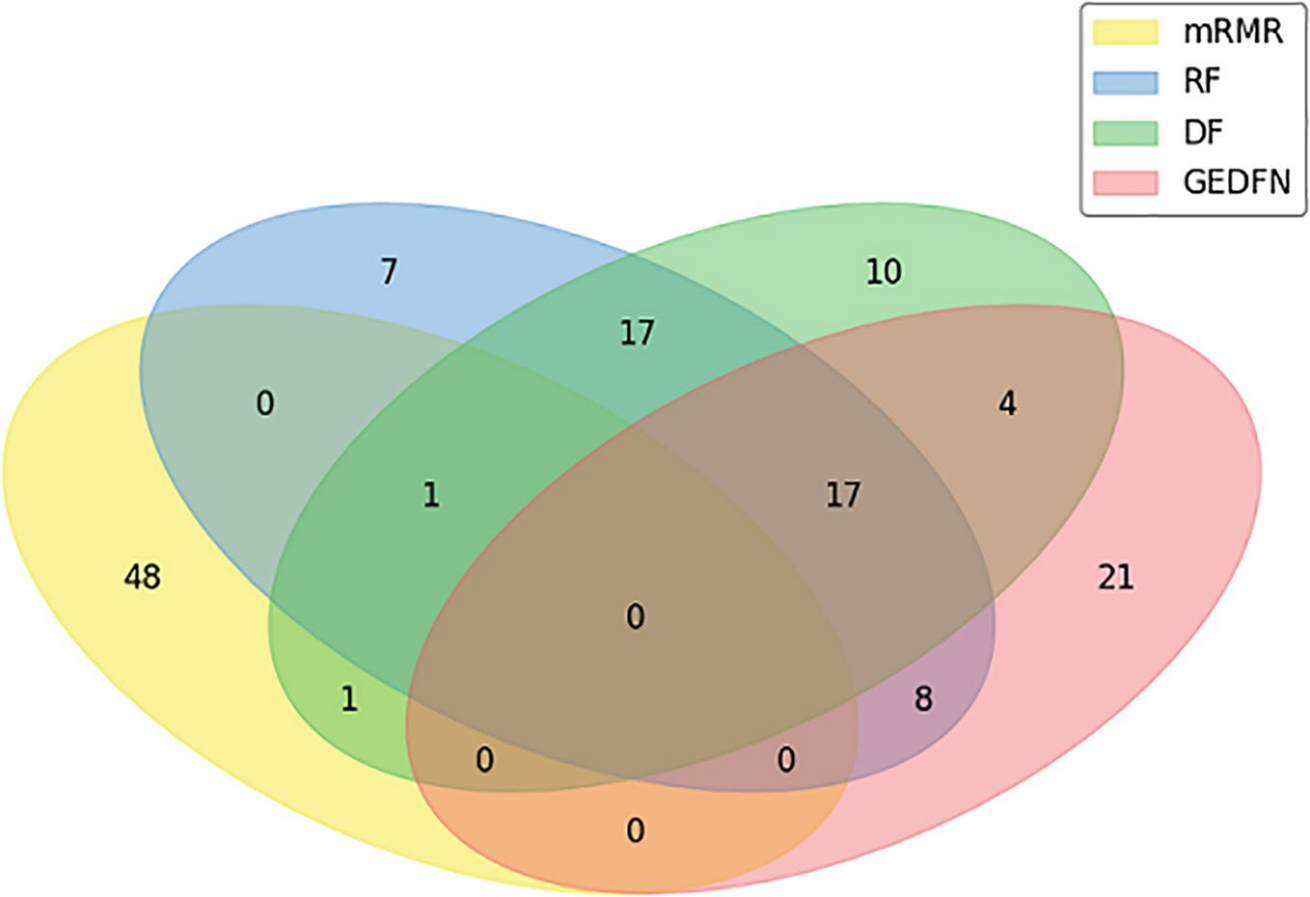
The feature selection based on Graph Embedding Deep Feedforward Network (GEDFN). The Venn diagram for top the 50 features selected *via* minimum Redundancy and Maximum Relevance (mRMR), Random Forest (RF), Deep Forest (DF) and GEDFN.

In our experiment, we compare GEDFN with traditional feature selection methods, such as minimum redundancy and maximum Relevance (mRMR) (Ding and Peng, 2005), Random Forest and Deep Forest respectively. Each method selects 50 features. We want to know if the features obtained by the traditional machine learning feature selection method can also be selected by GEDFN. As can be seen from the Venn diagram (**Figure 4**), most of the features selected by the mRMR are different from those selected by the other three methods. Among these 50 features selected by GEDFN, there are 25 and 21 features which are consistent with the Random Forest and Deep Forest respectively.

In addition, we compare the performance of GEDFN + SVM, RF + SVM, RF + SVM and RF + DF. Our approach is to select top 10, top 15, top 20,…, top 50 feature subsets from GEDFN and RF respectively, and test them on SVM and Deep Forest (DF) classifiers with five-fold cross-validation (**Table 1**). GEDFN + SVM, means GEDFN is utilized to conduct feature selection and SVM is the classifier. RF + SVM, means RF is utilized to conduct feature selection and SVM is the classifier. GEDFN + DF, means GEDFN is utilized to conduct feature selection and DF is the classifier. RF + DF, means RF is utilized to conduct feature selection and DF is the classifier.

**Table 1:** The performance among GEDFN + SVM, RF + SVM, GEDFN + DF and RF + DF.

#	GEDFN + SVM			RF + SVM			**GEDGN+DF**			**RF+DF**		
	P	R	F1	P	R	F1	P	R	F1	P	R	F1
10	0.733	1	**0.846**	0.675	1	0.806	0.733	1	**0.846**	0.785	0.871	0.825
15	0.745	1	**0.854**	0.675	1	0.806	0.745	1	**0.854**	0.722	0.909	0.800
20	0.752	1	**0.858**	0.675	1	0.806	0.750	0.991	0.854	0.717	0.927	0.805
25	0.706	1	0.828	0.675	1	0.806	0.705	0.991	0.824	0.765	0.907	**0.829**
30	0.707	1	**0.828**	0.675	1	0.806	0.707	0.983	0.823	0.718	0.957	0.821
35	0.698	1	**0.822**	0.675	1	0.806	0.698	1	**0.822**	0.692	0.977	0.810
40	0.704	1	**0.826**	0.675	1	0.806	0.709	0.985	0.824	0.706	0.962	0.813
45	0.707	1	**0.828**	0.675	1	0.806	0.707	1	**0.828**	0.687	0.991	0.811
50	0.697	1	**0.822**	0.675	1	0.806	0.697	1	**0.822**	0.695	0.974	0.810

#, number of top features; P, precision; R, recall; F1= $\frac{2\times P\times R}{P+R}$ . The best F1 scores are marked as bold.

From **Table 1**, the combination of GEDFN and SVM achieves the best f1 score, while RF + SVM gets the worst performance. Meanwhile, GEDFN + SVM and GEDFN + DF have consistent performance. We find GEDFN prefers the sparse features while RF prefers the dense features. In other words, RF has a bias in the feature selection process where multivalued features are favored (Nguyen et al., 2015). In addition, RF is biased in the presence of correlation and often identifies non-predictive features that are independent from each other (Nicodemus and Malley, 2009). Actually, the microbial data is sparse and the features are dependent, which makes RF not the best choice to conduct feature selection in microbiome. However, GEDFN is to embed the *priori* sparse correlation network and find biomarkers as a whole, which makes it more suitable for microbiome-wide association studies than RF-based models.

**Figure 5:**
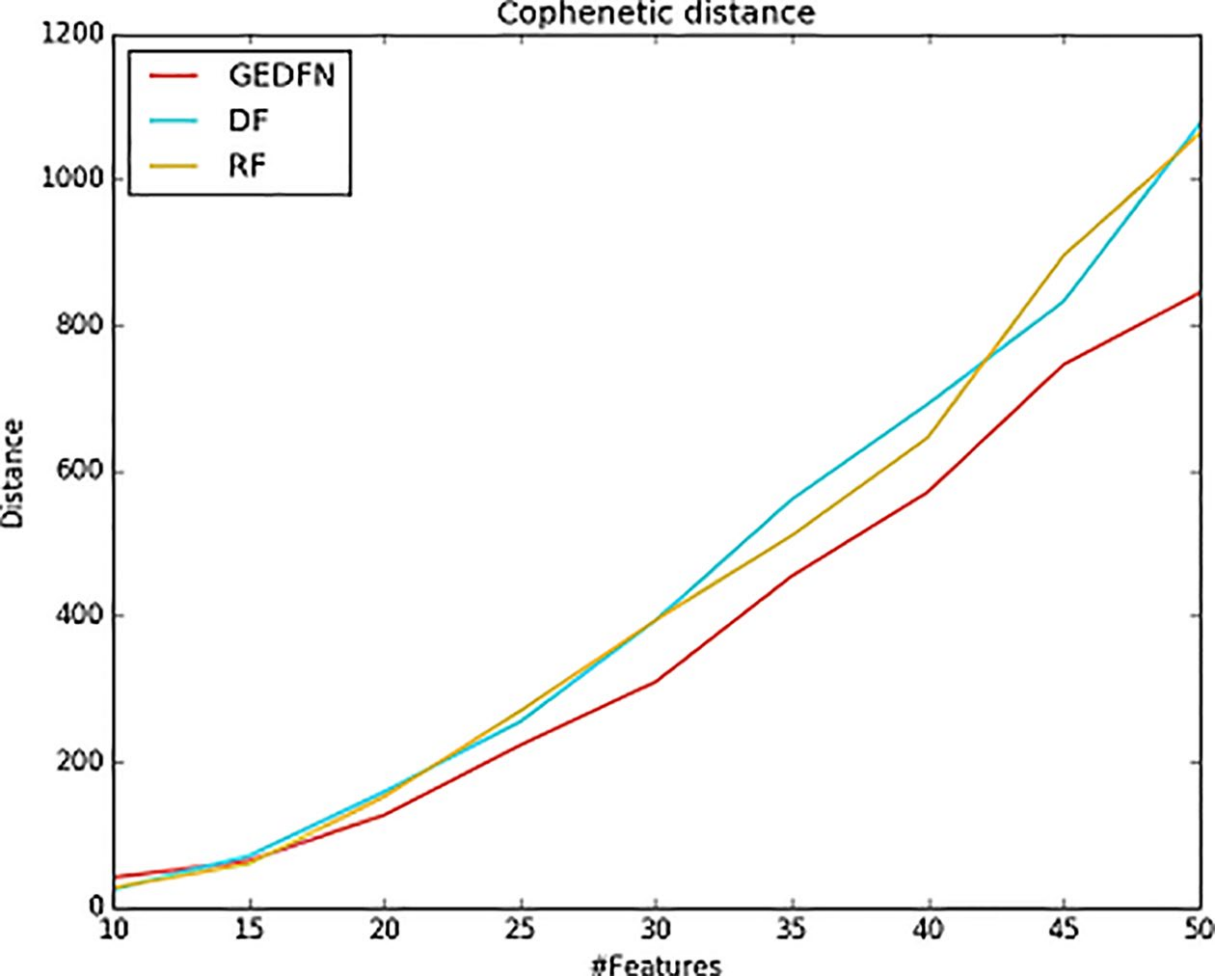
The cophenetic distance for top 50 features selected *via* Random Forest (RF), Deep Forest (DF) and Graph Embedding Deep Feedforward Network (GEDFN) respectively (The cophenetic distance is the sum of the features' pair-wise distance.). The cophenetic distance of two objects is a measure of how similar those two objects have to be in order to be grouped into the same cluster.

The cophenetic similarity or cophenetic distance of two objects is a measure of how similar those two objects have to be in order to be grouped into the same cluster (Sokal and Rohlf, 1962; Saraçli et al., 2013). We calculate the cophenetic distance of the feature subsets. The specific process is as follows: we select different feature subsets obtained by Random Forest, Deep Forest and GEDFN, such as top 10–50 features, and then calculate node-node pairwise distance. The distance is characterized by the leaf nodes of the phylogenetic tree. We use the cophenetic method of the ape package in R to calculate the node-node pairwise cophenetic distance. The value in the matrix is the sum of the branch lengths separating each pair of species. We compare the top 50 features of Random Forest, Deep Forest and GEDFN respectively. We find the feature subsets of GEDFN has smallest cophenetic distances among these methods, which means that the subset of these features is better cohesive and we speculate that this cohesion may be functional meaningful (**Figure 5**). Deep Forest and Random Forest have similar cophenetic distance because Deep Forest is a cascade structure based on Random Forest.

**Figure 6:**
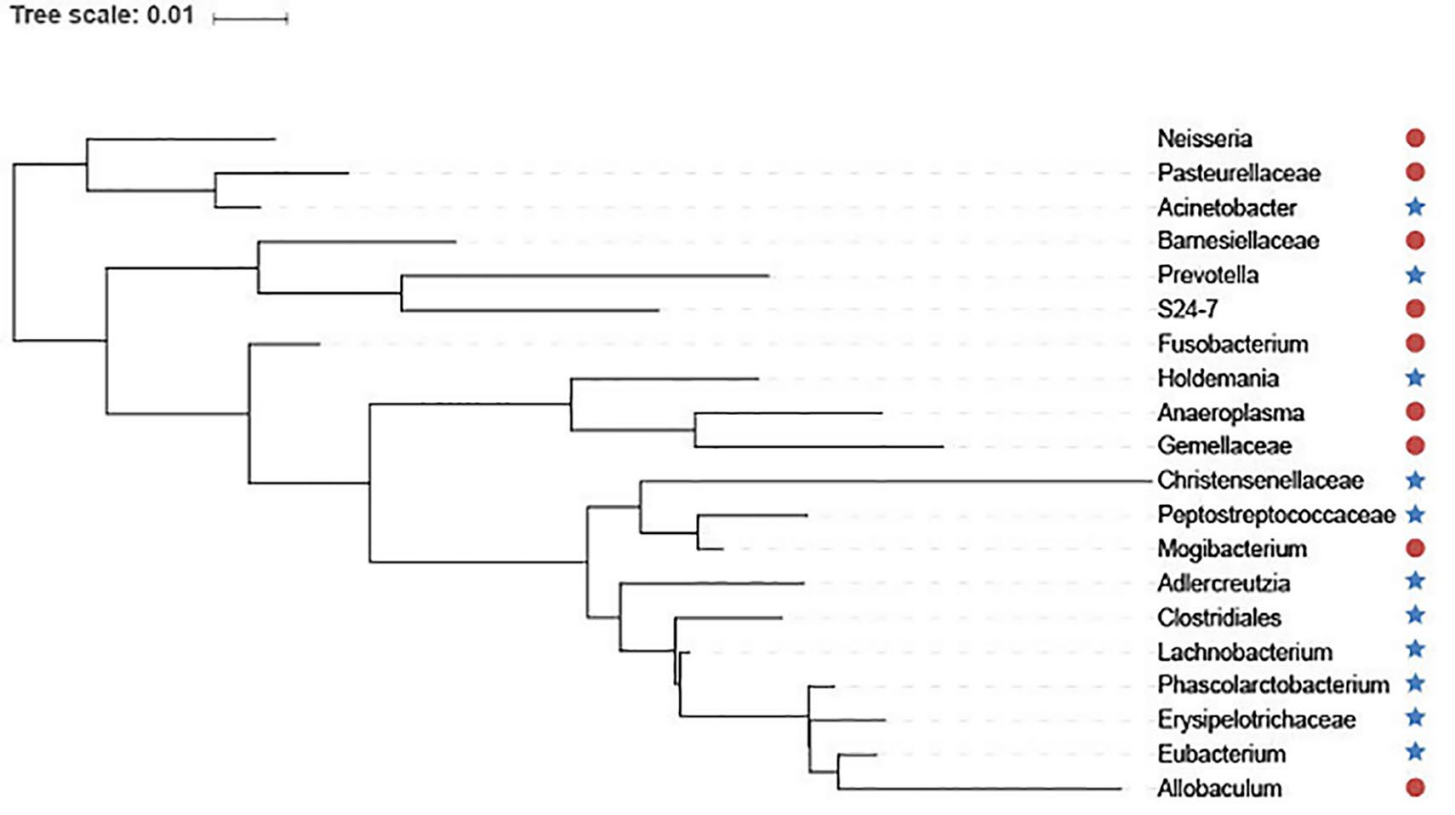
The top 20 species selected *via* Graph Embedding Deep Feedforward Network (GEDFN). The species in red circle are higher relative abundance while species in blue star are lower relative abundance in diseased group. These species are visualized on the phylogenetic tree.

In addition, we utilize interactive Tree Of Life (iTOL) (Letunic and Bork, 2016) to visualize the top 20 features selected by GEDFN (**Figure 6**). The features are ranked according to their importance score. We average each species' relative abundance for diseased and normal groups respectively. We find that *Neisseria*, *Pasteurellaceae*, *Bamesiellaceae*, *S24-7*, *Fusobacterium*, *Anaeroplasma* and *Gemellaceae* had high abundance compared to the normal group, while other microorganisms are lowly expressed in the disease group. The *Neisseria*, *Pasteurellaceae*, *Fusobacterium* and *Gemellaceae* increased in Crohn's disease, which was reported in the research (Gevers et al., 2014). The *Clostridiales*, *Eubacterium*, *Erysipelotrichaceae* and *Peptostreptococcaceae*, *Christensenellaceae* were found in lower relative abundance in Crohn's disease (Gevers et al., 2014; Matsuoka and Kanai, 2015; Pascal et al., 2017). However, there is no unified option on the Crohn's disease-related microbial biomarkers. As a result, our findings must need further experiments to explore and verify.

## Conclusions

In this work, we propose a method of embedding a microbial graph into a Deep Feedforward Network to achieve feature selection purpose. We have verified the feasibility of this method through experiments. The main contributions of our work are as follows: Firstly, the feasibility of this method is verified through combining microbial interaction structure and deep learning, and a sparse network structure is proposed. Secondly, the feature selection method is introduced into the microbial sparse network and the reliability of the feature selection results is verified, indicating that deep neural networks can also conduct feature selection. We hope our work will bring another perspective to the interpretability of deep learning.

The problems still exist in the research work. First of all, our work does not compare the influence of various methods of constructing microbial networks on feature selection (Weiss et al., 2016). The networks constructed by various methods are varying. We found that the reliability of the microbial network directly affected the subsequent results. Secondly, the threshold of association network was traded off and there was no relevant guidance suggestion. In general, the higher the threshold, the more reliable the network, but it would make the network too sparse. It would be required to balance the threshold and the network's sparseness. Finally, we only consider the influence of the weight parameters of the Deep Neural Network on the feature selection without considering the threshold of the neuron. Because it would involve the nonlinear transformation which could make the problem complicated and difficult. Therefore, our future work will focus on how to build a more reliable microbial interaction network and get more meaningful microbial markers.

## Data Availability Statement

The datasets generated for this study are available on request to the corresponding author.

## Author Contributions

Qiang Z, XJ and TH conceived the concept of the work and designed the experiments. Qing Z and MP performed literature search. Qing Z, XJ, MP and TH collected and analyzed the data. Qiang Z, XJ and MP wrote the paper. All authors have approved the final manuscript.

## Funding

This research is supported by the National Key Research and Development Program of China (2017YFC0909502) and the National Natural Science Foundation of China (No. 61532008 and 61872157).

## Conflict of Interest

The authors declare that the research was conducted in the absence of any commercial or financial relationships that could be construed as a potential conflict of interest.
