# Corrigendum: Graph Embedding Deep Learning Guides Microbial Biomarkers' Identification

**DOI:** 10.3389/fgene.2020.00487

**Published:** 2020-05-15

**Authors:** Qiang Zhu, Xingpeng Jiang, Qing Zhu, Min Pan, Tingting He

**Affiliations:** ^1^School of Information Management, Central China Normal University, Wuhan, China; ^2^School of Computer, Central China Normal University, Wuhan, China; ^3^Hubei Provincial Key Laboratory of Artificial Intelligence and Smart Learning, Central China Normal University, Wuhan, China

**Keywords:** graph embedding, deep learning, feature selection, biomarkers, microbiome

Although in the original article although we have cited the work (Kong and Yu, 2018) in the Introduction section, we did not cite the work in the Materials and Methods section. Our approach to embedding deep learning for identifying microbial biomarkers is based on their methods and thus contributed a lot to our article. Therefore, this citation has been added to the following sections.

In order to avoid misinterpretation, we would like to add the reference in the following places which were highlighted in RED:

The **Materials and Methods** section, subsection **The Framework of Graph Embedding Deep Feedforward Network**, sub-subsection **Graph Embedding Deep Feedforward Network**, paragraph 4:

“Where ⊙ is element-wise product. Therefore, the connection between the input and first hidden layer of the feedforward network is filtered by the graph adjacency matrix. Each feature is corresponding to a hidden neuron. All features have corresponding hidden neurons in the first hidden layer. The feature can only provide information to the connected graph. In this way, the graph helps to achieve the sparsity of the connection between the input layer and the first hidden layer (Kong and Yu, 2018).”

The **Materials and Methods** section, subsection **Feature Selection Based on GEDFN**, paragraph 1:

“In addition to improving classification, it is also meaningful to find features that contribute significantly to classification because they reveal potential biological mechanisms. However, Deep neural network is a “black box”, the interpretability of deep learning hasn't been well-defined (Guidotti et al., 2019). In our experiment, we focus on how the input features influence the prediction and we borrow the idea from Olden and Jackson (2002) and Kong and Yu (2018). The feature importance score is the quantification values of the contributions of features to a model prediction, which links the input features and output prediction. They highlight the parts of a given input that are most influential for the model prediction and thereby help to explain why such a prediction was made. The feature selection is based on feature score, which means the score is high if the feature is important. As a result, we develop a feature ranking method based on the feature relative importance score, similar to the connection weights method introduced by Olden and Jackson (2002) and Kong and Yu (2018). What is learned by neural networks is contained in the connection weights. Based on idea of connection weight, we propose a graphical connect weight method that emphasizes the importance of the features of our proposed neural network architecture.”

The authors apologize for this error and state that this does not change the scientific conclusions of the article in any way. The original article has been updated.
